# PAK-dependent regulation of actin dynamics in breast cancer cells

**DOI:** 10.1016/j.biocel.2022.106207

**Published:** 2022-05

**Authors:** Marianne Best, Madeline E. Gale, Claire M. Wells

**Affiliations:** a School of Cancer and Pharmaceutical Sciences, Kings College London, London UK; b North West Thames Regional Genetics Service, Northwick Park Hospital, London UK

**Keywords:** p21-activated kinases, Actin cytoskeletal dynamics, Breast cancer, Cell invasion, Metastasis, Migrastatics

## Abstract

Metastatic Breast Cancer has a poor 25% survival rate and currently there are no clinical therapeutics which target metastasis. ‘Migrastatics’ are a new drug class which target migration pathway effector proteins in order to inhibit cancer cell invasion and metastasis. The p21-activated kinases (PAKs) are essential drivers of breast cancer cell migration and invasion through their regulation of actin cytoskeletal dynamics. Therefore, the PAKs present as attractive migrastatic candidates. Here we review how PAKs regulate distinct aspects of breast cancer actin dynamics focussing on cytoskeletal reorganisation, cell:matrix adhesion, actomyosin contractility and degradative invasion. Lastly, we discuss the introduction of PAK migrastatics into the well-honed breast cancer clinical pipeline.

## Highlights

• The p21-activated kinases (PAKs) reside at a nexus of cell signalling pathways which promote breast cancer metastasis.
• PAK isoforms employ distinct functional roles specifically regulating actin dynamics to drive breast cancer cell invasion.
• Administration of PAK migrastatics with current anti-proliferative treatment could suppress breast cancer metastasis.

## 1 Introduction

**Fig. 1:**
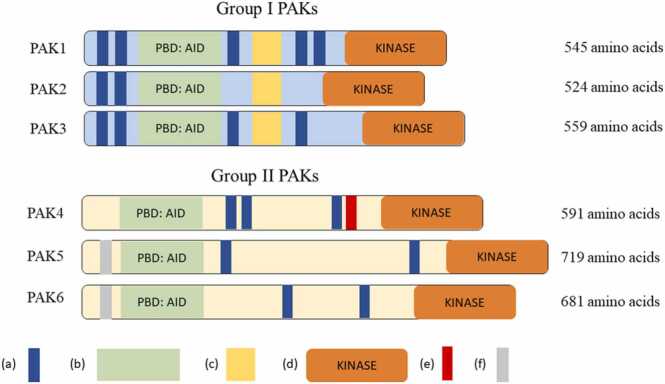
The p21-activated kinase (PAK) domains. The PAK family has six isoforms categorised into Group I PAKs and Group II PAKs. PAKs differ between their groups as well as between isoforms. Each subdomain is shown with the isoform amino acid length. (a) Proline rich region, (b) PID / AID, (c) PIX binding site, (d) kinase domain, (e) Rho-GEF interacting domain, (f) Nuclear localisation signal. Abbreviations are PBD = p21-binding domain, AID = Autoinhibitory domain, and PIX = PAK-interacting exchange factor and GEF = Guanine exchange factor.

Breast Cancer (BCa) is the most common cancer in women, with females having a 12% lifetime risk of developing the disease. Stage 1 BCa has a remarkable 98% 5-year survival rate, however this drops to 25% for metastatic Stage 4 BCa, partly explained by a gap in clinical strategy to treat metastasis (Cancer Research UK, 2021). Migrastatics propose to transform the current therapeutic landscape by providing a selective way to disrupt cancer cell migration and metastatic colonisation at distal sites (Rosel et al., 2019). A cellular pre-requisite for metastasis is adoption of a migratory phenotype (Williams et al., 2019). This is dependent on the dynamic reorganisation of the actin cytoskeleton, predominantly delivered by Rho family GTPases Rac1, Cdc42 and RhoA, through a tight network of downstream effector proteins (King et al., 2014). One notable effector family are the p21-activated kinases (PAKs) which are positioned at the nexus of numerous signalling pathways controlling actin cytoskeletal protrusions, matrix adhesions, actomyosin contractility and invadosome-driven degradation of extracellular matrix (ECM) (King et al., 2014). This family of serine/ threonine kinases are highly conserved across a wide range of organisms and are categorised into two groups based on domain homology and mechanisms of regulation (Fig. 1). PAKs 1–3 comprise Group I PAKs and PAKs 4–6 comprise Group II PAKs (Bokoch, 2003). Their centralised roles in numerous oncogenic signalling pathways and frequent overexpression and/ or amplification in human cancers, notably PAK1 and PAK4 in BCa, lend themselves as ideal therapeutic candidates (King et al., 2014, Rane and Minden, 2019). Traction is building for PAK migrastatic development and the use of migrastatics alongside anti-proliferative therapy for BCa could prevent both growth and development of secondary metastases (Anderson et al., 2018).

## 2 The role of PAKs in breast cancer cell migration and invasion

The regulation of actin dynamics orchestrates cancer cell migration and invasion. Firstly, actin fibre polymerisation and stability drive initial membrane extension at the leading edge. Secondly, adhesion of the extended actin cytoskeleton to the ECM, followed by generation of contractile forces, pull the cell body forward (Pellegrin and Mellor, 2007). Next, actin-rich invadopodia degrade ECM by secreting matrix metalloproteinases (MMPs), facilitating cell invasion through tissue architecture. Actin filament recycling and disassembly of invadopodia and adhesion complexes is a crucial final step to allow for continuation of cell invasion (Ayala et al., 2008, Williams et al., 2019).

### 2.1 PAK and actin reorganisation

The cell periphery maintains high actin monomer density, primed for actin polymerisation and generation of protrusive structures, like lamellipodia and filopodia. Both PAK1 and PAK4 can induce actin polymerisation through LIM-kinase (LIMK) activity which phosphorylates cofilin to inactivate and inhibit its actin severing ability, allowing actin filament stabilisation and growth (Edwards et al., 1999, Wang et al., 2007). PAK1 can also regulate cofilin dephosphorylation to disrupt actin polymerisation and impede lamellipodia formation, suggesting that PAK1 can bypass LIMK and likely activate a cofilin phosphatase instead (Coniglio et al., 2008). Interesting in this scenario, depletion of PAK2 did not alter cofilin phosphorylation levels (Coniglio et al., 2008), and a similar parallel between PAK1 and PAK2 has been shown in mast cells (Kosoff et al., 2013), whereby these isoforms have opposing roles in actin reorganisation and degranulation. PAK1-induced rearrangement of the actin cytoskeleton promotes mast cell degranulation, yet PAK2 instead negatively regulates degranulation through RhoA inhibition (Kosoff et al., 2013). Of interest, it has been reported that serine protease tryptase, the main component of mast cell cargo, is correlated with poor BCa prognosis (Aponte-López et al., 2018). These data suggest that PAK1 and PAK2 could be working antagonistically in a feedback loop, yet no evidence demonstrates a direct link between the two isoforms. More likely, PAK1 and PAK2 exert opposing effects onto the same biological outcome, which could be explained by cell localisation, substrate differences and/or differences in activating signals. Antagonistic roles for kinases are not unusual; they are important for generating balanced signal outputs, yet also add a layer of complexity for kinase inhibitor design. A recent publication identified sphingosine kinase 2 (SphK2) in driving PAK1 towards cofilin phosphorylation to promote triple negative BCa (TNBC) cell invasion (Gupta et al., 2021). Similarly, indirect inhibition of phospho-PAK1 via Nimbolide treatment of TNBC cells enhanced levels of unphosphorylated cofilin, inducing actin depolymerisation (Arumugam et al., 2021). TNBC cells may depend on PAK1 for actin polymerisation and cell invasion, meaning isoform-specific inhibitors could benefit this subtype; however, comparison of other PAK isoforms/ BCa subtypes were not made.

Stabilisation of polymerised actin occurs through generation of a branched actin network. The seven-subunit actin related protein-2/3 complex (Arp2/3) provides nucleation sites for *de novo* actin filaments at a 70° angle from the original, exerting a pushing force and initiating cell membrane extension. p41-Arc, a subunit protein of Arp2/3 was found to be regulated by PAK1 in BCa cells (Vadlamudi et al., 2004). MCF-7 cells expressing a p41-Arc mutant (with point mutation, T21A) could not localise p41-Arc to regions of actin polymerisation, inhibiting 2D migration (Vadlamudi et al., 2004). A protein interactome study also using MCF-7 cells identified that PAK4 interacts with N-WASP, a critical actin-binding regulator, to phosphorylate Ser^484^/Ser^485^ and promote Arp2/3-dependent actin branching (Zhao et al., 2017). Despite being implicated in BCa progression, the role of PAK5 and PAK6 in BCa actin polymerisation/ stability remains to be explored.

### 2.2 PAK and stress fibre-associated cell adhesions

Metastasising BCa cells must balance actomyosin contractility and formation of cellular adhesions to drive propulsive cell migration. Stress fibres (SF) are contractile actomyosin bundles, composed of 10–30 actin filaments held together by actin cross-linking protein, α-actinin (Pellegrin and Mellor, 2007). Located at the ends of SF are focal adhesions (FA), subcellular complexes required to mechanically link intracellular actin to extracellular matrix, as well as deliver integrin-mediated signalling predominantly via Rho GTPase-activation of downstream effector proteins (Pellegrin and Mellor, 2007) i.e., PAKs. There is limited information regarding PAK isoform-specific roles in cellular adhesion. Most PAK inhibitors target PAKs by group, and kinase-dead mutants may be dominant against all PAK isoforms and not recapitulate true conditions. To further complicate this, it has been speculated that overexpression of proteins can override isoform specificity. Overexpression of a kinase-dead PAK1 in the highly metastatic MDA-MB-435 BCa cell line exhibited SF containing thick F-actin filaments, an increased number of FA, dramatic enhancement of cell spreading and a 3-fold reduction in cell invasion (Adam et al., 2000). A comparative study of PAK1 and PAK2 in T47D BCa invasion used siRNA knockdowns to discriminate between isoform function and showed that PAK2 regulated both FA size and generation by distinct mechanisms (Coniglio et al., 2008). PAK2 limits FA size through inhibition of RhoA, yet its regulation of FA generation is independent of RhoA, potentially reflecting a direct requirement for PAK2 here. In contrast to the link between PAK1 activity and FA number reported by Adam et al., the authors demonstrated that PAK1-depletion had no effect on FA number and instead increased FA size and showed FA were unable to mature (Coniglio et al., 2008). Knockdown studies also come with their caveats: full interpretation can be difficult with the potential for redundancy or secondary effects from the abundance of GTPases; therefore, drawing comparisons between studies using distinct techniques should be done with caution. Despite distinctions regarding PAK1 and FA number, what these studies do make clear is that both PAK1 and PAK2 play essential roles in BCa cellular adhesion. Furthermore, there is evidence to suggest that PAK4 plays a kinase-independent role in BCa FA turnover, PAK4-mediated adhesion dynamics did not require kinase activity nor interaction with Cdc42, and instead was controlled through a novel PAK4-RhoU interaction (Dart et al., 2015). Thus, Group I PAKs could be more associated with adhesion formation/maturation and Group II PAKs with adhesion turnover, although further clarification is needed for PAK5 and PAK6 in BCa cell adhesion function.

### 2.3 PAK and myosin light chain regulation

Actomyosin contractility depends upon actin interaction with myosin in the myosin light chain (MLC) pathway. MLC-kinase (MLCK) phosphorylates myosin-II which binds to actin filaments and generates contractile forces countered by SF and FA (Coniglio et al., 2008). Differential functions were also reported for PAK1 and PAK2 in T47D BCa cells. PAK1 knockdown decreased phospho-MLCK levels, while PAK2 knockdown enhanced MLCK phosphorylation. The aforementioned PAK2-driven RhoA inhibition was found to feedback onto MLCK modulation, revealing a novel PAK2/RhoA/MLC axis in BCA cellular adhesion (Coniglio et al., 2008).

### 2.4 PAK and degradation of ECM through invadopodia

Invadopodia are actin-rich cell protrusions reliant upon actin dynamics for their turnover. They mediate invasion by secreting MMPs to enzymatically degrade ECM and it is thought that BCa cells utilise invadopodia for successful metastatic dissemination (Williams et al., 2019). Invadopodia formation and structure is well-characterised and while it is understood that Rho GTPases are key invadopodia regulators, less is known about the downstream molecular mechanisms regulating actin dynamics (Williams et al., 2019). Moshfegh et al. (2014) reported that PAK1 phosphorylation of cortactin at Ser^113^ regulated invadopodia disassembly via a Trio-Rac1-PAK1 signalling axis in metastatic BCa cells, and not in epithelial breast cells. It was proposed that p27 acts by promoting the cortactin/PAK1 interaction and phosphorylation; however this work was not performed in BCa cells (Davis et al., 2017). Corroborating these findings, a separate study demonstrated that actin puncta generation (nascent invadopodia) was not supressed in PAK1-knockdown BCa cells, yet these cells exhibited increased levels of ECM degradation (Williams et al., 2019). Further investigation revealed that PAK1 impacts cofilin and MLC phosphorylation directly within invadopodia during disassembly, driving turnover and cell invasion (Williams et al., 2019).

Interestingly, a study in melanoma cells suggested that PAK1 regulated invadopodia formation (Ayala et al., 2008), intriguingly via the same Ser^113^ phosphorylation site as Moshfegh and colleagues reported, sparking debate over whether PAK1-regulation of invadopodia actin dynamics is cancer-type specific. Further studies in melanoma also supported a role for PAK1-dependent invadopodia formation, whereby PAK1-depleted melanoma cells could not initiate actin puncta (Nicholas et al., 2016). Evidently, PAK1 has a key function in invadopodia actin dynamics, but it remains to be seen if these PAK1 distinctions are true for the heterogeneous spectrum of BCa subtypes. Currently, the majority of published studies use TNBC cell lines, hence further investigation with BCa panels will be necessary to tease out distinctions that could guide PAK therapeutic design. There is also the question of PAK isoform-specific differences in invadopodia regulation. PAK1 and PAK2 are contrasting in their regulation of actin polymerisation and cellular adhesion, yet there is currently no direct evidence that PAK2 regulates invadopodia activity in BCa. Also, a novel role for PAK4 in invadopodia maturation has been reported in melanoma cells (Nicholas et al., 2016), yet how this translates to BCa has yet to be elucidated.

There are increasing reports of PAK5 implicated in BCa progression, yet still minimal data exploring its role in invadopodia. In BCa, MMP2 is trafficked to invadopodia leading edges by cortactin to degrade ECM; a study found that in comparison to controls, PAK5-depleted MDA-MB-231 and BT459 BCa cells exhibited reduced in vitro migration and invasion as well as downregulated expression of MMP2 in cell lysates (Wang et al., 2013). To the best of our knowledge a role for PAK6 in BCa invadopodia actin dynamics is not reported.

## 3 PAK inhibitors as breast cancer migrastatics

**Fig 2:**
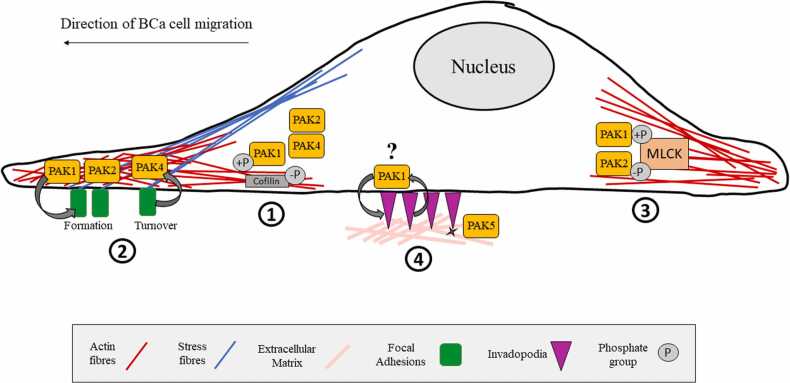
p21-activated kinase (PAK) regulation of Breast Cancer cell actin dynamics. Schematic showing PAK-dependent actin dynamics which promote Breast Cancer cell migration and invasion. (1) Polymerization and stabilization of actin fibres which can be regulated by PAK1 phosphorylation and dephosphorylation, depending on conditions. Also, PAK2 and PAK4 function here. (2) Cell adhesion by stress fibre formation and focal adhesions which counter membrane tension. PAK1 and PAK2 are linked to focal adhesion formation, whereas PAK4 is associated with turnover. (3) Actomyosin contractility is regulated by PAK1 phosphorylating MLCK, and PAK2 dephosphorylating MLCK in order to affect generation of contractile forces. (4) Degradative invasion through invadopodia. The role of PAK1 in invadopodia formation or lifetime is debated, and it is unknown what the role of other PAKs are here. Evidence points towards a role of PAK5 in promoting MMP secretion from invadopodia for ECM degradation. Abbreviations are MLCK = myosin light chain kinase, MMP = matrix metalloproteinase.

Major improvements in cancer patient survival over the past few decades are in part due to advances made in selective/targeted therapy, immunotherapy and cancer screening programmes (Anderson et al., 2018). Unfortunately, improvement in these survival rates does not equally reflect those with metastatic cancer. Until recently, a prerequisite for cancer treatment was tumour shrinkage, highlighting how the end point of therapeutic design is still focussed on the primary tumour (Rosel et al., 2019). We have reviewed how PAKs are implicated in BCa cell actin dynamics to modulate cytoskeletal extensions, cell adhesion, actomyosin contractility, and degradative invasion to overall promote BCa metastatic dissemination (Fig. 2). The pursuit to develop PAK inhibitors as cancer migrastatics has intensified, although no PAK inhibitors have yet made it into clinic (Radu et al., 2014, Yao et al., 2020).

Pfizer compound PF-3758309, initially designed to be a PAK4-specific ATP-competitive inhibitor, disrupted PAK4-dependent cell adhesion and anchorage-independent growth in MDA-MB-231 xenograft tumour models, corresponding to an 89% reduction in BCa tumour growth (Murray et al., 2010). However, it was found that PF-3758309 acted upon both Group I and II PAKs, as well as off-target kinases, rendering it more broad-spectrum than PAK4-specific. The striking effect on tumour growth by PF-3758309 drove its progression to Phase 1 trials, only for it to be later withdrawn from clinical use due to undesirable pharmacological properties (Table 1) (Murray et al., 2010, Radu et al., 2014) . Similarly, ATP-competitive inhibitor FRAX597, which is a non-specific Group I PAK inhibitor, also bound off-target kinases (Semenova and Chernoff, 2017). The highly conserved PAK family kinase domain means it is difficult to develop ATP-competitive kinase inhibitors that are isoform-specific and avoid off-target binding. Therefore, allosteric small molecule inhibitors are gaining traction as an alternative due to precision targeting resulting in fewer off-target effects, however these inhibitors tend to be less potent (Semenova and Chernoff, 2017). IPA-3 targets the PAK1 isoform through its regulatory domain (Deacon et al., 2008) and inhibits human MDA-MB-231 and mouse 4T1 cell migration and invasion, without affecting viability (Khan et al., 2020). IPA-3 treatment had a stronger inhibitory effect on the 4T1 cells as PAK1 is expressed at higher levels than the MDA-MB-231 (Khan et al., 2020), showing the direct effect PAK1 has on BCa invasion and how a personalised therapeutic approach could match isoform-selective migrastatics to tumours with isoform specific expression patterns.

**Table 1:** p21-activated kinase (PAK) inhibitors.

Name of PAK-inhibitor	Type	Intracellular Targets
PF-3758309(Pfizer)	ATP-competitive	Initially reported as PAK4-specific, yet binds both Group I and II PAKs.Also binds off-target kinases: AMP-dependent kinase and ribosomal S6 kinase.
FRAX597(Cayman Chemical)	ATP-competitive	Group I PAK inhibitor, with selectivity for PAK1 inhibition.Also binds off-target kinases: RET, YES1, TEK, CSF1R.
IPA-3	Allosteric small molecule inhibitor	Group I PAK inhibitor, with selectivity for PAK1 inhibition.
Nimbolide	Neem *(Azadirachta indica)* plant-derived triterpene	Multiple targets, including Rac1/Cdc42 activity, indirectly inhibiting PAKs.

The clinical pipeline in BCa is well-honed, making the introduction of PAK migrastatics especially challenging. BCa cells may become invasive early in cancer development and can metastasise to liver, lung, brain and bone, although preference is distinct between BCa subtypes (Molnár et al., 2017). Therefore, prevention of secondary metastases is a plausible rationale for migrastatic intervention (Anderson et al., 2018). A challenging but crucial objective will be the consolidation of migrastatics with other therapies; this is likely to be as an adjuvant medicine to surgery and other standard-of-care therapies, with the potential for follow-up ‘maintenance therapy’ to prevent recurrence after initial treatment of the primary tumour (Anderson et al., 2018). Indeed, the use of PAK migrastatics as long-term maintenance therapy will need to address the risk of chronic toxicities and the lowest dose should be identified (Anderson et al., 2018). PAK migrastatics may be also useful in preventing treatment-induced metastasis or could be given as neoadjuvant to surgery. Targeting cytoskeletal actin dynamics has the potential to affect leukocyte and other non-malignant cell migration (Rosel et al., 2019); however, as PAKs are overexpressed and/or amplified in cancer compared to the relatively low levels in normal tissue and do not mutate (Rane and Minden, 2019), this could circumvent the issue. Although PAK4 at least plays a key role in heamatopoietic cell adhesion (Foxall et al., 2019). Indeed, disruption of the actin cytoskeleton from PAK migrastatic adminstration could increase drug-sensitisitivy of cancer cells demonstrating the potential of synergy with current anti-proliferative medicine (Rosel et al., 2019, Trendowski, 2014). The regulatory pathway is open and PAK migrastatic development is already underway, yet we cannot dismiss the further characterisation that is needed for less-well known PAK isoforms. The potential for PAK isoform-selective inhibitors designed for BCa subtypes is a challenging, yet highly rewarding goal. We believe it is the right time to tackle this and enter a new era of anti-metastatic therapeutics.
